# Global Electroencephalography Synchronization as a New Indicator for Tracking Emotional Changes of a Group of Individuals during Video Watching

**DOI:** 10.3389/fnhum.2017.00577

**Published:** 2017-12-01

**Authors:** Chang-Hee Han, Jun-Hak Lee, Jeong-Hwan Lim, Yong-Wook Kim, Chang-Hwan Im

**Affiliations:** Department of Biomedical Engineering, Hanyang University, Seoul, South Korea

**Keywords:** electroencephalography (EEG), affective brain-computer interface (aBCI), passive brain-computer interface, global field synchronization (GFS), neurocinematics

## Abstract

In the present study, we investigated whether global electroencephalography (EEG) synchronization can be a new promising index for tracking emotional arousal changes of a group of individuals during video watching. Global field synchronization (GFS), an index known to correlate with human cognitive processes, was evaluated; this index quantified the global temporal synchronization among multichannel EEG data recorded from a group of participants (*n* = 25) during the plays of two short video clips. The two video clips were each about 5 min long and were designed to evoke negative (fearful) or positive (happy) emotion, respectively. Another group of participants (*n* = 37) was asked to select the two most emotionally arousing (most touching or most fearful) scenes in each clip. The results of these questionnaire surveys were used as the ground-truth to evaluate whether the GFS could detect emotional highlights of both video clips. The emotional highlights estimated using the grand-averaged GFS waveforms of the first group were also compared with those evaluated from galvanic skin response, photoplethysmography, and multimedia content analysis, which are conventional methods used to estimate temporal changes in emotional arousal during video plays. From our results, we found that beta-band GFS values decreased during high emotional arousal, regardless of the type of emotional stimulus. Moreover, the emotional highlights estimated using the GFS waveforms coincided best with those found by the questionnaire surveys. These findings suggest that GFS might be applicable as a new index for tracking emotional arousal changes of a group of individuals during video watching, and is likely to be used to evaluate or edit movies, TV commercials, and other broadcast products.

## Introduction

Understanding human emotional processes is an important research topic in neuroscience, since emotion plays a key role in the communications and interactions among people. A number of studies have aimed at estimating human emotional state using bio-signals. Traditionally, human emotional state has been robustly estimated by extracting features from facial expressions and speech sounds (Black and Yacoob, 1997; Petrushin, 1999; Anderson and McOwan, 2006). Although these modalities have proven to be useful for evaluating and classifying emotional state, they are not applicable in nonverbal environments or in the absence of cameras. Other studies have used peripheral physiological signals, such as electrocardiogram, skin conductance, and respiration signals to recognize current emotional states of individuals. However, since some of these signals exhibited relatively slow responses (e.g., heart rate variability index requires at least 1-min delay), the features extracted from them could not be effectively used to monitor immediate emotional changes. More importantly, these signals are affected not only by changes in emotional state, but also by other independent factors such as stress, physical fatigue, and alcohol intake. Thus, the recognition of human emotional state from such bio-signals is generally regarded to be difficult without constructing an individually customized “ground-truth” database for each specific emotion (Kim et al., 2004; Wang et al., 2014). As an alternative approach, Kim et al. (2004) showed that brain signals could be used for more robust estimation of emotional state because brain signals potentially reflect human emotional state more directly than the physiological responses. Among various neuroimaging modalities, such as electroencephalography (EEG), magnetoencephalography (MEG), and functional magnetic resonance imaging (fMRI) that have been used to reveal the neural substrates of emotion (Peyk et al., 2008), EEG has been considered a most suitable tool to study the dynamics of emotion due to its excellent temporal resolution and reasonable cost (Millán et al., 2008). Recently, decoding of emotional state from EEG has attracted increased attention because of the popularization of low-cost wearable EEG systems and their potential applications in affective brain-computer interfaces (aBCIs).

Two methods, frontal alpha-asymmetry and event-related potentials (ERPs), have been most widely used to evaluate or recognize human emotional state using EEG (Davidson, 1992; Baumgartner et al., 2006; Degabriele et al., 2011; Petrantonakis and Hadjileontiadis, 2011). Frontal alpha-asymmetry, which quantifies asymmetry in the alpha-band powers recorded over the left and right frontal lobes, has been extensively used as an indicator of emotional arousal in humans despite its variability from individual to individual (Davidson, 1992; Baumgartner et al., 2006; Petrantonakis and Hadjileontiadis, 2011). Alternatively, the responses of ERP components to different emotional stimuli have also been studied (Degabriele et al., 2011). However, most studies based on these methods have focused on recognizing overall changes in emotional state in response to limited emotional stimuli such as sounds, tastes, and pictures. Only a few studies have attempted to monitor continuous emotional change using EEG (Dmochowski et al., 2012, 2014; Soleymani et al., 2015). Estimation of emotional changes elicited by multimedia contents in a group of individuals can potentially be applied to the evaluation of various cultural contents, including movies, TV commercials, and music videos; however, reliably tracking the continuous emotional change is still a challenging issue, and thus more effective and sensitive features need to be developed.

In this study, we focused on developing a new index by which temporal changes in emotional state of a group of individuals can be precisely estimated. To this end, we recorded and analyzed physiological responses of a number of participants while they were watching video clips designed to evoke contrasting emotions. Among the potential indices, we tested the feasibility of global EEG synchronization as an emotional state index. This choice was based on the observation that functional connectivity is generally increased during cognitive processing, whereas it is decreased during emotional processing, especially in the beta and gamma frequency bands (Northoff et al., 2004; Harding et al., 2012). Beta-band global field synchronization (GFS), a well-known measure which have been shown to correlate strongly with the human cognitive process (Koenig et al., 2001; Lee et al., 2010), was used to quantify the global synchronization of multichannel EEG data recorded from a group of participants. We hypothesized that stimuli eliciting high emotional arousal would lead to decreased GFS values because dominant emotional processing, also referred to as *flow* in the field of psychology, might distract from cognitive processing in the brain. To validate whether the GFS can be effectively used as an index for tracking temporal changes of emotion in a group of individuals, we compared the grand-averaged GFS waveforms with ground-truth data obtained from questionnaire surveys as well as the estimates of emotional arousal obtained using conventional methods including galvanic skin response (GSR), photoplethysmography (PPG), and multimedia content analysis (MCA).

## Materials and methods

### Subjects

Two different groups of subjects were recruited for this study. The first group consisted of 25 healthy participants (19 males, 6 females, mean age 24.04 ± 3.13 years) and the second group consisted of 37 participants (25 males, 12 females, mean age 24.32 ± 2.00 years). All participants had normal or corrected-to-normal vision and none had a previous history of neurological disease, psychiatric disease, or any other severe disease that could have affected the study results. Before the experiments, detailed written and verbal information on the experiments was given to all participants, who received monetary reimbursement for their participation in our study. This study was carried out in accordance with the recommendations of the Institutional Review Board (IRB) of Hanyang University with written informed consent from all subjects. All subjects gave written informed consent in accordance with the Declaration of Helsinki. The protocol was approved by the IRB of Hanyang University.

### Experimental conditions

Two short video clips were presented to the participants. One video clip, which was used to arouse happiness emotion, was a short video clip entitled “Isaac's Live Lip-Dub Proposal.” This clip had been watched by more than 30 million people on YouTube (http://youtu.be/5_v7QrIW0zY) as of 2016 and will hereafter be referred to as the “positive clip.” In the video, which is 5 min 13 s long, a man named Isaac surprises his girlfriend with the “world's first” live lip-dub marriage proposal. The other clip, which was used to elicit fearful emotion, had been edited from a horror movie entitled “The Grudge.” In this video, which is 4 min 36 s long, sudden appearances of ghosts made subjects feel fearful. This video clip will hereafter be referred to as the “negative clip.” A 17-inch LCD monitor was used to play the two video clips. The participants were seated in a comfortable armchair placed in front of the monitor, with the distance between the monitor and the participants set to 70 cm.

### Experimental paradigm

**Figure 1:**
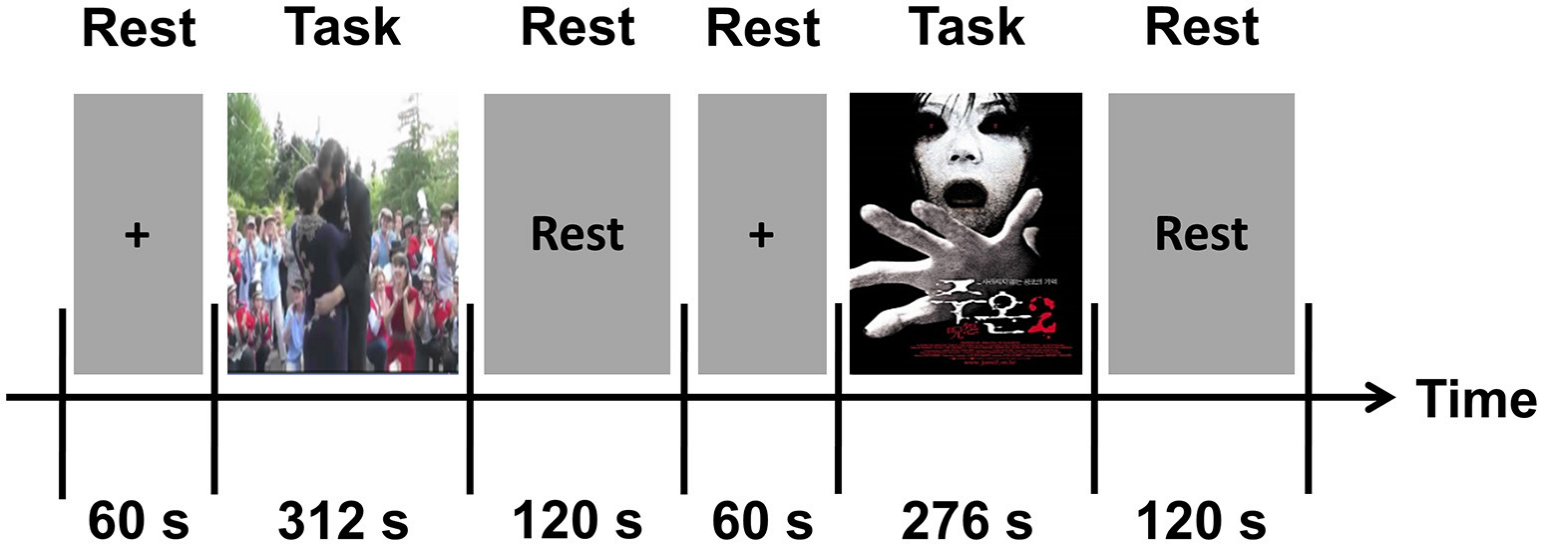
A schematic diagram of our experimental paradigm.

Participants in the first group (*n* = 25) watched each video clip while EEG, GSR, and PPG signals were recorded from them. Figure 1 shows a schematic diagram of our experimental paradigm. Before starting the experiment, a 60-s resting period was provided to each participant. The positive clip was presented first; after a 2-min rest, the negative clip was presented. This order was chosen because the positive clip was much less “emotionally stimulating” to the participants than the negative clip, and thus was likely to less affect the results of the following experiment. The audio portion of each clip was delivered using earphones.

To find the “ground-truth” about the temporal changes in emotional arousal of each video clip, the same video clips were presented to the participants of the second group (*n* = 37), except that no EEG, GSR, and PPG signals were recorded. The study participants watched each video clip twice. For the first viewing, participants were asked simply to concentrate on the video. At the conclusion of each video clip, participants were asked to memorize the two most emotionally arousing (most touching or most fearful) scenes in each clip. They were asked to select two scenes in each video clip because participants had difficulty selecting more than two impressive scenes in such short video clips. Then, during the second viewing, the participants were asked to record these two-time points on a questionnaire. The elapsed time was included in the video clips during the second viewing so that the participants could record the exact time information. In our study, the two subject groups were separately enrolled in two independent experiments. This design allow us to investigate whether emotional arousal changes estimated from one group can be generally applied to other groups of individuals, by which the general applicability of the proposed index might be validated.

### Data acquisition

EEG signals were recorded using a multichannel EEG system (ActiveTwo, BioSemi, Netherlands) with a head-cap mounted with active electrodes arranged according to the international 10–20 system. E-Prime software (Psychology Software Tools, Pittsburgh, PA, USA) was used to accurately synchronize the video start times during EEG recording. EEG signals were recorded from 22 scalp positions (Cz, C3, C4, T7, T8, Pz, P3, P4, P7, P8, Fp1, Fp2, Fz, F3, F4, F7, F8, AFz, AF7, AF8, O1, and O2). GSR and PPG data were recorded using the same system (ActiveTwo). Two GSR electrodes were adhered to each participant's left index and middle fingers, and a PPG sensor was attached to one's right index finger. Additionally, two electrodes were used to measure the horizontal and vertical electrooculograms (EOGs). One electrode was placed below the right eye for vertical EOG recording and the other electrode was placed at the outer canthus of the right eye for horizontal EOG recording. All data were recorded at a sampling rate of 2,048 Hz. The ground was composed of a feedback loop between the common mode sense (CMS) active electrode and the driven right leg (DRL) passive electrode, to which the closest approximation is a POz electrode.

### EEG analysis: GFS computation

Raw EEG data were preprocessed to remove unwanted artifacts. All preprocessing procedures were conducted using Curry 7 Neuroimaging software (Compumedics USA, Inc., Charlotte, NC, USA). The raw EEG data were re-referenced with a common average reference. We removed DC components in all channels by subtracting the mean of time series of each channel, and then the data were bandpass-filtered at 1 and 55 Hz cutoff frequencies. EOG artifacts by eye blinks were removed using principal component analysis. Finally, the preprocessed EEG data were segmented into 2-s epochs with a 50% overlap to continuously evaluate beta-band (14–25 Hz) GFS values over time. The GFS time-series of each participant was averaged across all participants, and then the grand-averaged GFS time-series was filtered using a ten-point moving-average filter. Moving average filtering was used because it was difficult to recognize the peak positions in the GFS waveforms due to the high-frequency ripples present in the raw GFS waveforms.

Global field synchronization, a method used to quantify the overall functional connectivity of the brain, has generally been used to investigate cognitive decline in patients with psychiatric disorders (Koenig et al., 2001; Lee et al., 2010). To determine the GFS values, EEG signals recorded from different scalp locations are first transformed into frequency domains using the Fast Fourier Transform (FFT), and then the FFT-transformed signals at each frequency are mapped onto a complex plane. Hanning window was used for each of the 2-s epochs before applying the FFT. The GFS value of a frequency is defined as the normalized difference between the two eigenvalues that represent the point distributions of the FFT results obtained from all channels in the 2-D complex plane (Koenig et al., 2001). The GFS is defined as

$$GFS\left(f\right)=\;\frac{\left|E{\left(f\right)}_{1}-E{\left(f\right)}_{2}\right|}{E{\left(f\right)}_{1}+E{\left(f\right)}_{2}},$$

Where *E*(*f*)_1_ and *E*(*f*)_2_ are the two eigenvalues obtained from principal component analysis at a given frequency, *f*. A GFS value close to 1 can be interpreted as increased overall functional connectivity. On the other hand, a GFS value close to 0 represents decreased overall functional connectivity. Lee et al. (2010) showed that the average GFS value in the beta band (14–25 Hz) is strongly correlated with the Mini-Mental States Examination (MMSE) score, which is known to reflect cognitive functioning in the brain.

### GSR and PPG analysis

GSR and PPG signals have been well-known that they are closely associated with changes of emotional arousal (McCurdy, 1950; Lang, 1995) because they contain information on the status of the autonomic nervous system (Kim et al., 2004; Kim and André, 2008). In this study, temporal changes in the emotional features obtained from GSR and PPG (Kim et al., 2004; Kim and André, 2008) were compared with GFS waveforms and ground-truth data.

**Figure 2:**
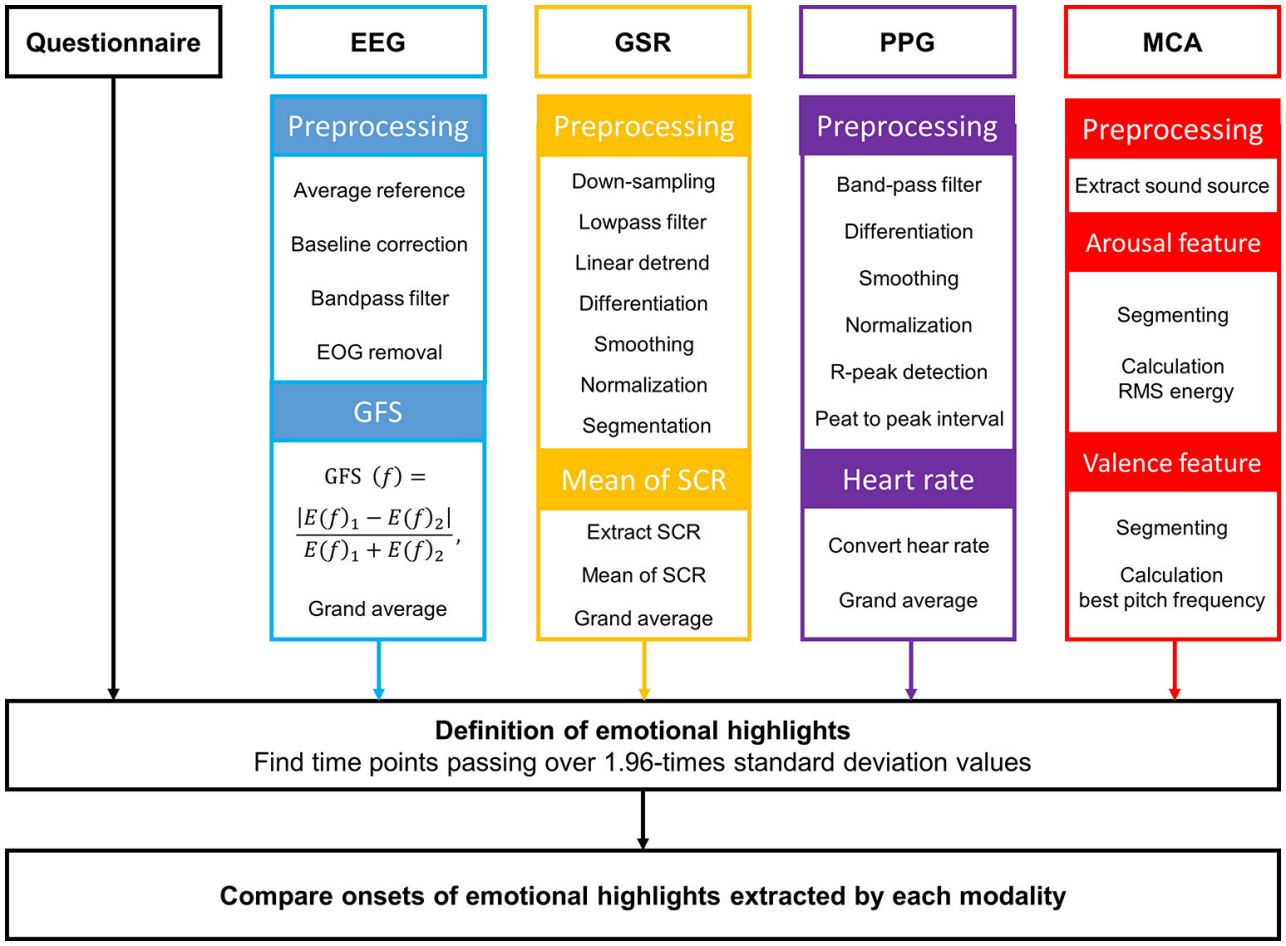
Overall procedure of data analysis.

The raw GSR data recorded during video watching were preprocessed using the following procedure: down-sampling, low-pass filtering, linear detrending, differentiation, smoothing, normalization, and segmentation (see Figure 2). Raw GSR data were down-sampled from 2,048 to 16 Hz and lowpass-filtered at a 0.2 Hz cutoff frequency using a third-order Butterworth zero-phase filter. To obtain the GSR data without DC components, linear detrending was applied. Slope values in the detrended data were then highlighted by the first-order differentiation. The first derivatives were smoothened by a subsequent convolution with a 20-point Bartlett window. The smoothed values were normalized by z-score transformation. In order to extract an emotional feature from the GSR data, the preprocessed data were segmented using a moving window of 6 sec. The distinctive short waveforms, which are called skin conductance responses (SCRs), are one of sensitive emotional indices obtainable from GSR. The occurrence of the SCRs in each epoch was detected by finding two consecutive zero-crossings, from negative to positive and from positive to negative. Then, the mean of maximum peaks in the SCRs was calculated and was used as the emotional feature of GSR in this study. Finally, the time-series of this GSR feature for each participant were grand-averaged across all participants, and this grand-averaged waveform was filtered using a ten-point moving-average filter.

The PPG data recorded during video watching were preprocessed using the following procedure: band-pass filtering, differentiation, smoothing, normalization, and segmentation. Raw PPG data were bandpass-filtered at 0.5 and 5 Hz cutoff frequencies using a third-order Butterworth zero-phase filter. First-order differentiation and subsequent convolution with a 20-point Bartlett window were performed to highlight slope values and to smooth the data, respectively. A z-score transformation was conducted to normalize the preprocessed data. Using the preprocessed data, heart rate (HR) was evaluated for each individual. We extracted time points of maximum peaks of all spikes in each PPG waveform and calculated peak to peak intervals (PPIs). The consecutive PPIs could be readily transformed to an HR time-series; each HR time-series were then grand-averaged across all participants. This grand averaged HR waveform was filtered using a ten-point moving-average filter.

### Multimedia content analysis (MCA)

Multimedia content analysis (MCA) is used to automatically evaluate the semantic meanings of a multimedia document (Wang et al., 2000). Previous studies have attempted to extract affective cues in multimedia contents using the MCA. For example, a previous study proposed a method for searching highlights in sports TV broadcasts that had been expected to excite the users most (Hanjalic and Xu, 2005). In the present study, we applied the MCA to our data in order to estimate streams of emotional change in our video clips. Features extracted from the MCA were compared with the results of GFS, PPG, GSR, and the questionnaire survey.

We extracted sound sources from each video clip. According to the reports of previous studies, it is well-established that energy a sound source is closely related with emotional arousal (Rui et al., 2000; Hanjalic, 2003; Hanjalic and Xu, 2005). The root mean square (RMS) energy was evaluated using the *mirpitch* function implemented in the MIR toolbox (Lartillot and Toiviainen, 2007).

## Results

**Figure 3:**
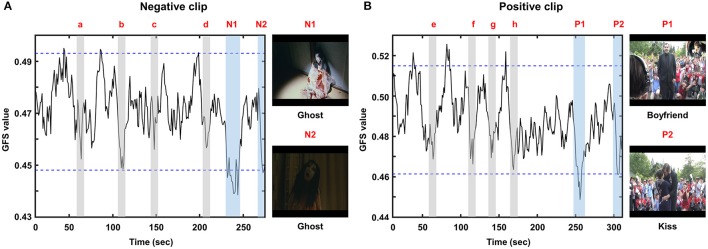
Grand averaged GFS waveforms: **(A)** negative clip; **(B)** positive clip. N1, N2, P1, and P2 represent time periods during which the GFS values dropped below the lower horizontal line. Additional time periods during which the GFS values suddenly dropped near the lower horizontal line (a, b, c, d, e, f, g, and h) are also marked as gray areas.

**Figure 4:**
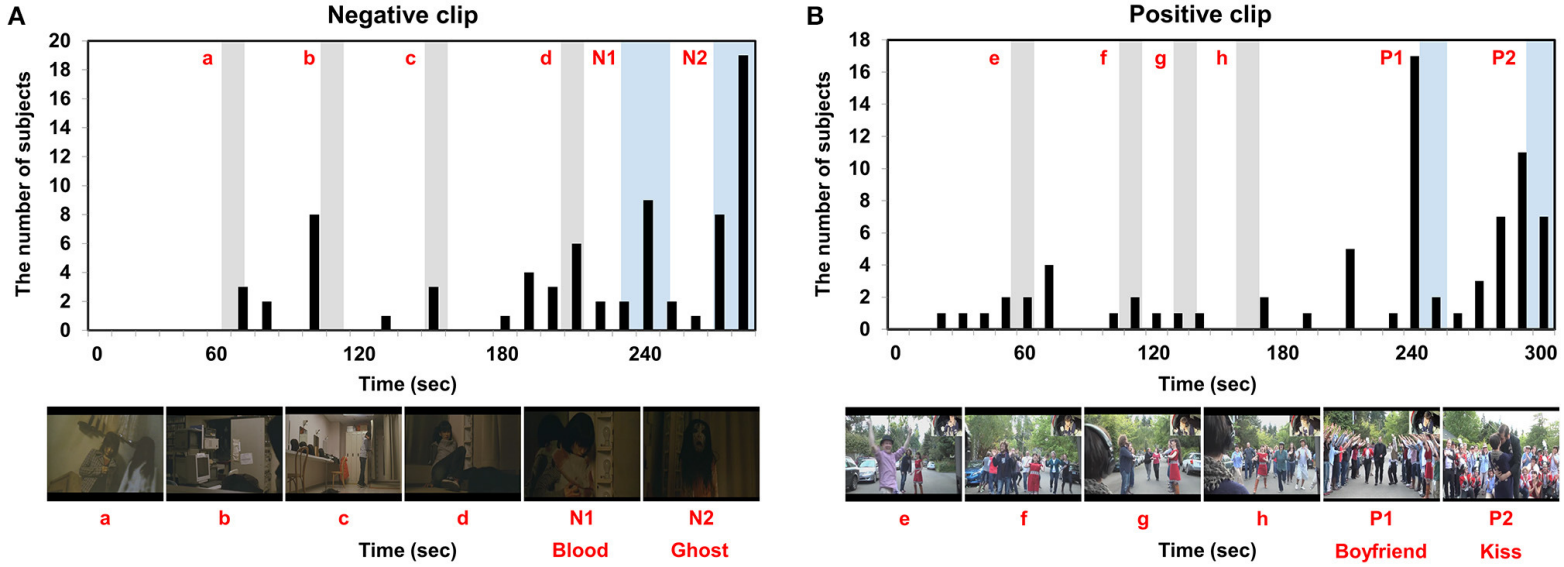
Two most impressive scenes in each clip as identified in the questionnaires: **(A)** negative clip; **(B)** positive clip. N1, N2, P1, and P2 represent time periods during which the GFS values dropped below the lower horizontal line. Additional time periods during which the GFS values suddenly dropped near the lower horizontal line (a, b, c, d, e, f, g, and h) are also marked as gray areas.

The grand-averaged GFS waveforms of each video clip are shown in Figure 3, whereas the two most impressive or memorable scenes in each clip as assessed by the other group of participants are shown in Figure 4. The blue horizontal lines in Figure 3 represent 1.96-times the standard deviation values (*p* = 0.05). Interestingly, the two-time points in each video clip at which the GFS values dropped below the lower horizontal line (N1, N2, P1, and P2) matched well with the time points most frequently chosen as impressive or memorable by the participants in the second group. At N1, the sudden appearance of a ghost and a character covered with blood made the audience feel most fearful. At N2, the ghost rushed to the character and a close-up of the ghost's face was shown. At P1, a female received a marriage proposal from her boyfriend right after a sudden appearance of him. At P2, her boyfriend kissed her. Additional time points at which the GFS values suddenly dropped near the lower horizontal line (a, b, c, d, e, f, g, and h) are also shown in Figure 3; some participants also reported high emotional arousal at these time points, as shown in Figure 4. At a, b, c, and d, frightening events occurred such as the sudden appearance of ghosts or the sudden drop of a wig (see the still images in Figure 4A). At e, f, g, and h, a group of humorous characters appeared and danced together (see the still images in Figure 4B). The full movie clips, along with the GFS waveforms over time, are attached as supplementary movie files (Supplementary1.wmv: positive clip; Supplementary2.wmv: negative clip). High resolution movie clips can be found at YouTubeTM (http://youtu.be/-fT_FYZ2Ceo–positive clip; http://youtu.be/N-dPAAtkhss–negative clip).

To rule out the possibility that the GFS waveform changes were skewed by some participants, the GFS values averaged for time points marked as emotional events in Figure 3 (e.g., P1, P2, a, b, c, d, etc.) were compared with the GFS values averaged over the time points not marked as emotional events using the two-tailed paired *t*-test for all participants' data. This analysis revealed that the GFS levels over the two conditions were significantly different (positive clip: *p* = 0.0068; negative clip: *p* = 0.0059). The GFS waveforms displayed with standard errors are also included in the Supplementary Information File. In addition, to exclude the possibility that the GFS waveforms were affected by muscle artifacts generally contaminating EEG signals at temporal electrodes (T7 and T8), we first evaluated variations of beta-band power spectral densities (PSD) at T7 and T8, the results of which are presented in the Supplementary Information File (Figures S2a,b). No noticeable correlations between the beta-band PSDs and the GFS waveforms were found. In addition, we excluded the two temporal electrodes (T7 and T8) and used only 20 electrodes to evaluate the GFS waveforms. The results are also presented in the Supplementary Information File (Figure S2c). It could be seen from the figure that the overall trends of GFS waveforms were not changed after the two electrodes had been excluded from the analysis. These results demonstrate that the GFS waveforms were not affected by the muscle artifacts recorded at the temporal EEG channels.

The mean GFS values exhibited small variability from individual to individual. The average GFS values for the positive and negative clips were 0.488 ± 0.026 and 0.470 ± 0.015, respectively. The standard deviations of both GFS values were much less than the dynamic ranges of the grand-averaged GFS waveforms (Figure 3). The average GFS value in the positive clip (0.488) was slightly larger than that in the negative clip (0.470) (Figure 3); however, they did not exhibit any significant difference (*p* = 0.297, two-tailed paired *t*-test).

**Figure 5:**
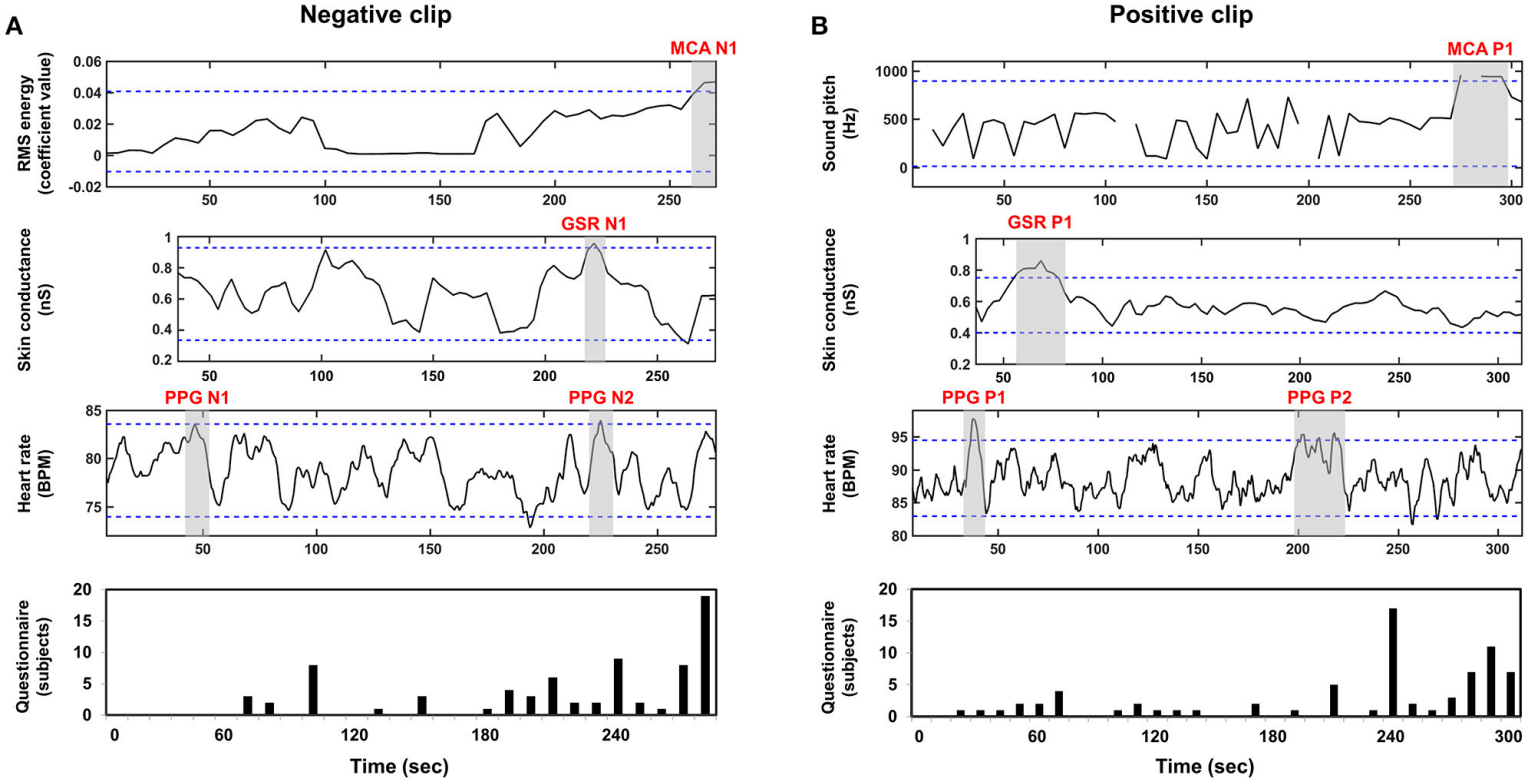
Grand averaged waveforms of MCA, GSR, PPG, and questionnaires results: **(A)** negative clip; **(B)** positive clip; First, second, and third row represent MCA, GSR, and PPG waveforms, respectively. A fourth row shows the results of the questionnaire survey.

We compared other emotional features evaluated using MCA, GSR, and PPG with the results of questionnaire survey and GFS in order to demonstrate the superiority of the GFS. Figure 5 shows the grand-averaged waveforms of the additional features from MCA, GSR, and PPG. The blue horizontal lines in each figure represent 1.96-times standard deviation (*p* = 0.05). In the results of MCA, time intervals showing highest RMS energy, denoted by MCA N1 and MCA P1 for the negative and positive clips, respectively, coincided fairly well with the time periods most frequently chosen by the participants of the second group (see Figure 4). In the GSR results, a time period denoted by GSR N1 in the SCR waveform for the negative clip matched well with N1 period in Figure 4; while the only peak appearing in the SCR waveform for the positive clip, which is denoted by GSR P1, did not match with any peaks in Figure 4. No peaks in the HR waveforms (PPG N1, PPG N2, PPG P1, and PPG P2) coincided with the results of the questionnaire survey. These results demonstrated that the GFS was the only reliable index that could precisely estimate the emotional highlights.

## Discussion

This study aimed to track continuous changes in emotional arousal in a group of individuals using GFS, a measure of global EEG synchronization. In support of our hypothesis, decreased GFS values were observed when stimuli eliciting high emotional arousal were presented. It is particularly noteworthy that decreased GFS values were observed regardless of the stimulus valence. This finding supports our hypothesis that emotional processing distracts from cognitive processing, and that the GFS value, an indicator of cognitive processing, decreases during emotional processing. The scenes identified as most impressionable/memorable matched fairly well with the grand-averaged GFS waveforms, suggesting that our study design can potentially be applied to practical applications, such as the evaluation of cultural content or broadcasting products.

Only a few studies have attempted to track continuous changes in brain status while subjects watch videos (Dmochowski et al., 2012, 2014; Kong et al., 2013). In a study by Kong et al., general global field power was evaluated. This metric was defined as the weighted sum of the alpha-band power and theta-band power, and was based on the assumption that alpha-band and theta-band powers are associated with attention and memorization, respectively. An empirical index, named the impression index, coincided well with questionnaire results. However, the index values were highly dependent on the powers of specific rhythmic activities which generally exhibit significant inter-individual and intra-individual variability, making it difficult to define the most appropriate threshold at which the video clips should be evaluated. Moreover, since the impression index was defined using attention and memorization, emotional arousal changes could not be detected using this index. In two studies by Dmochowski et al. (2012, 2014), maximally correlated components were extracted from EEG signals recorded from multiple participants. Temporal changes of these components were then compared with the corresponding movie scenes. However, since the index in this study was based on a data-driven approach rather than a hypothesis, the index is likely to be dependent on the particular participant population and cannot be described as an explicit equation. Our approach, which is based on global EEG synchronization, differs from the previous studies in the following aspects: (1) In contrast to the study by Kong et al. (2013), we adopted a normalized global EEG synchronization approach that is known to have small inter-individual variance among healthy subjects (Lee et al., 2010). Thus, our method facilitates the direct evaluation of the video clip impressions on the subjects. (2) Since our index can be expressed as an explicit equation, it can be readily applied to other movie clips without any modifications. (3) The impression index proposed by Kong et al. could not differentiate cognitive processes from emotional processes. Therefore, the use of multiple indices including ours would likely enhance the overall reliability of EEG-based evaluation of video clips. Future studies will focus on investigating this topic.

A series of studies have demonstrated that cortical functional connectivity has strong positive correlations with cognitive load and cognitive ability. For example, the execution of specific mental tasks requiring a high cognitive load, such as working memory tasks, oddball tasks, and stroop tasks, resulted in significant increases of overall functional connectivity (Polich et al., 1986; MacLeod, 1992; Hampson et al., 2006; Kim et al., 2008), particularly in the beta and gamma frequency bands. In other studies, beta-band global functional connectivity, which is frequently measured using GFS, has been reported to have a strong negative correlation with cognitive decline in patients with Alzheimer's disease or individuals with cognitive impairments (Kikuchi et al., 2007; Czigler et al., 2008; Lee et al., 2010). Therefore, the decreased beta-band GFS might indicate that the individuals' brains were not involved in high-load cognitive processing. In this study, we hypothesized that the grand averaged GFS value would decrease when stimuli eliciting high emotional arousal were presented to participants, because emotional processing could potentially distract from cognitive processing in the brain. Although this hypothesis could be tested by comparing the GFS waveforms with the questionnaire results, the scenes at which increased GFS values were observed could not be quantitatively evaluated due to the lack of an appropriate measurement. We plan to pursue the development of such quantitative measures in future studies.

In our study, we divided participants into two groups and only recorded EEG data from one group. We separated the “training” group from the “test” group in order to demonstrate the general applicability of our index. Similar experiments were conducted recently by Dmochowski et al. (2014). In their study, the EEG impression index evaluated from a few audiences was compared with the ratings of much larger audiences. They found that the ratings of the larger audience could be predicted quite accurately by the EEG index obtained from the small number of individuals who had participated in the experiments. In our study results, the temporal changes in GFS values of a group of participants coincided well with the questionnaire survey results acquired from another group of participants, demonstrating the expandability of our new index. For example, the tracking of the emotional index such as the GFS during movie screening might be used for film-editing. During the film editing processes, the producers or film-makers should arrange scenes or shots into appropriate places (Cutting et al., 2010) because appropriate positioning of shots significantly contributes to the viewers' various emotional responses such as attention and immersion. Accordingly, time-resolved tracking of emotional responses via bio-signals would help the film-makers to find the most appropriate places to arrange shots.

## Author contributions

C-HH: wrote literature, completed literature review; performed analysis of MCA, GSR, and PPG; main contributing author. Ju-HL: wrote literature, analyzed EEG data; main contributing author. C-HI: final approval authority, guided overall study process, advanced concepts of this study; significant contribution to authorship. Je-HL: advised in fundamental concepts, assisted our experiment. Y-WK: assisted our experiment, advised about process of data analysis.

### Conflict of interest statement

The authors declare that the research was conducted in the absence of any commercial or financial relationships that could be construed as a potential conflict of interest.
