# Supplementary material for: Global Electroencephalography Synchronization as a New Indicator for Tracking Emotional Changes of a Group of Individuals during Video Watching
Source: Front Hum Neurosci. 2017 Dec 1;11:577. doi: 10.3389/fnhum.2017.00577 (PMC5717022; doi:10.3389/fnhum.2017.00577)
Supplement: Supplementary file 3 [file Image1.PDF]

Supplementary Figures

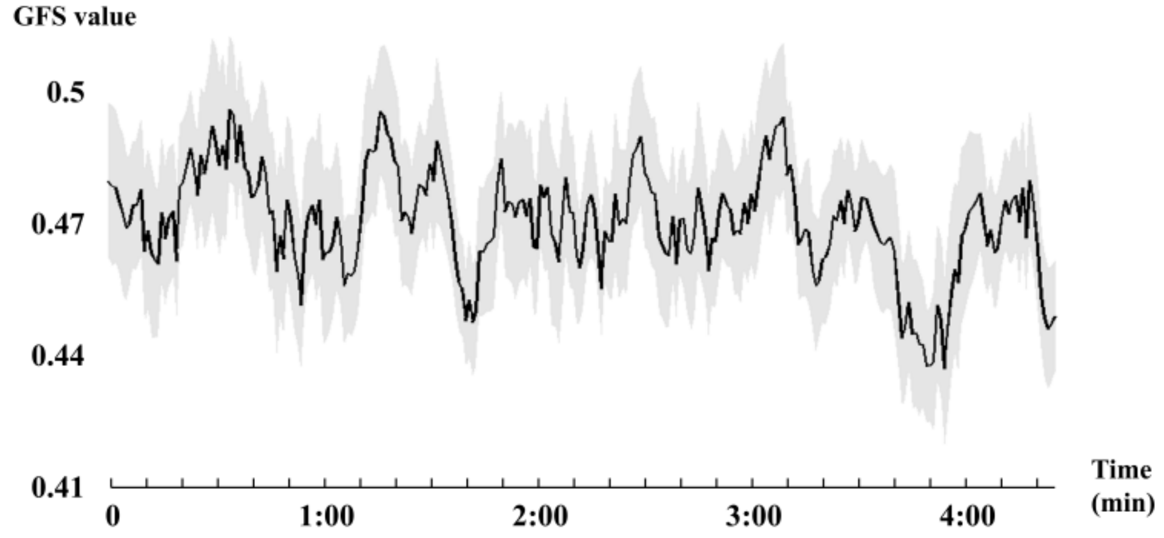

(a)

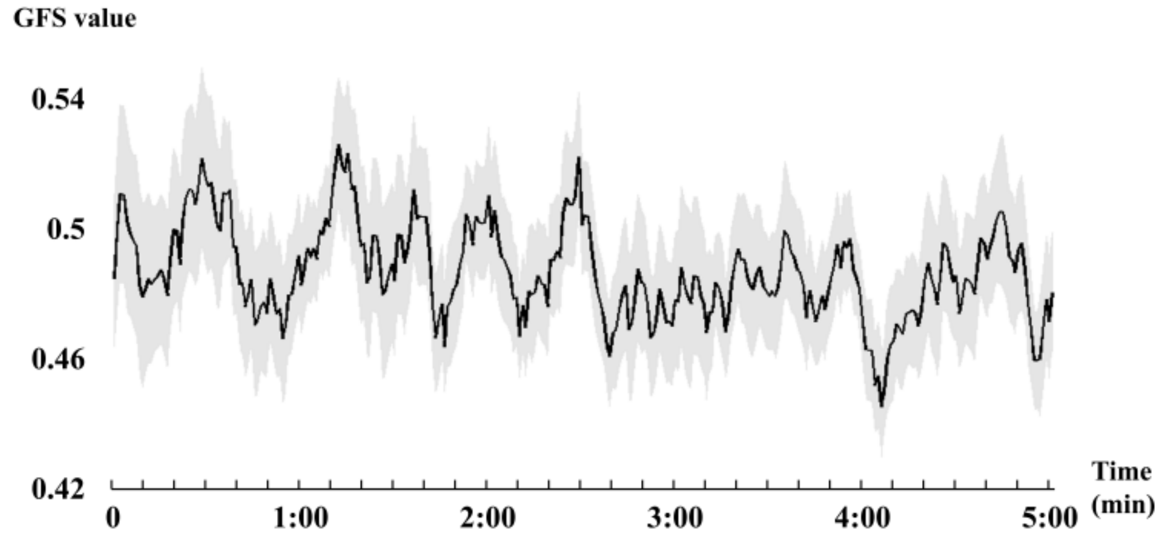

(b)

Figure S1. GFS waveforms with standard errors: (a) GFS waveform during negative clip watching, (b) GFS waveform during positive clip watching

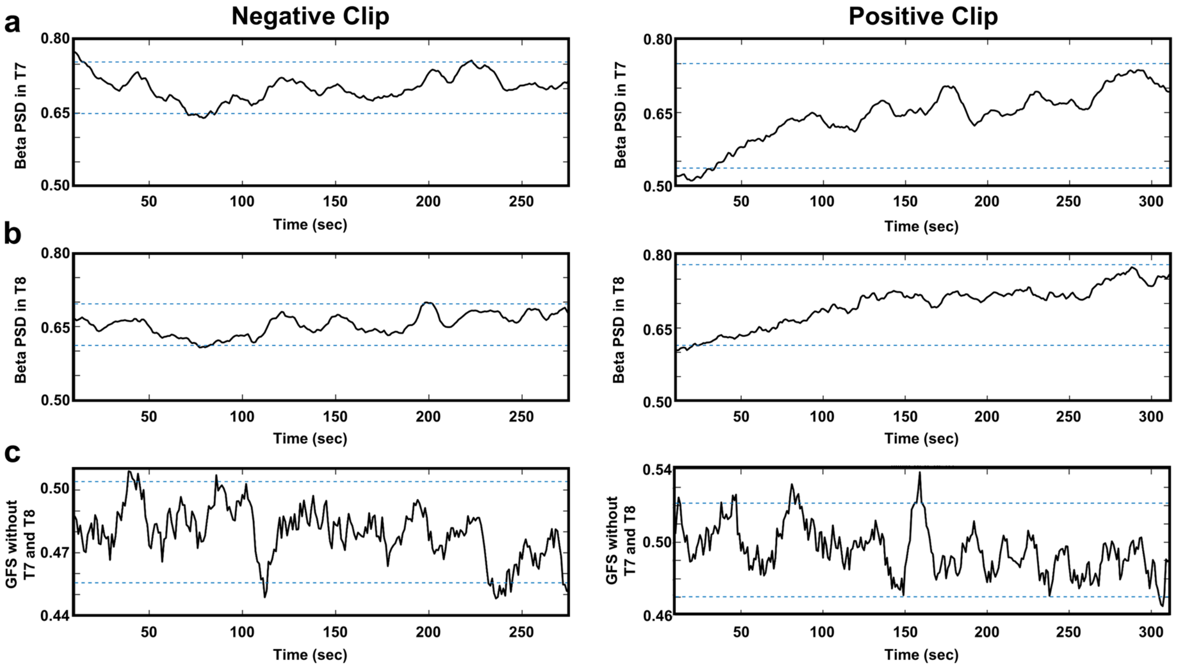

Figure S2. (a) Time-varying changes of beta-band power spectral density (PSD) at T7. Left and right graphs show the beta PSD waveforms for the negative-clip and positive-clip conditions, respectively. (b) Time-varying changes of beta-band PSD at T8. Left and right graphs show the beta PSD waveforms for the negative-clip and positive-clip conditions, respectively. The variations of beta PSD at temporal electrodes did not show noticeable correlations with the GFS waveforms. (c) The GFS waveforms evaluated after excluding both temporal electrodes (T7 and T8), which shows that the overall trend of GFS was not changed.
